# Preoperative Diquafosol vs. Intense Pulsed Light with Gland Expression for MGD: Effects on Refractive Accuracy and Tear Film Stability in Cataract Surgery

**DOI:** 10.3390/jcm14248946

**Published:** 2025-12-18

**Authors:** Takeshi Teshigawara, Tatsukata Kawagoe, Yuki Mizuki, Miki Akaishi, Takuto Sakono, Kazuro Yabuki, Seiichiro Hata, Akira Meguro, Nobuhisa Mizuki

**Affiliations:** 1 Department of Ophthalmology, Tsurumi Chuoh Eye Clinic, Yokohama 230-0051, Japan; 2 Department of Ophthalmology, Yokosuka Chuoh Eye Clinic, Yokosuka 238-0008, Japan; 3 Department of Ophthalmology, Yokohama City University School of Medicine, Yokohama 236-0004, Japan; kawagoe@yokohama-cu.ac.jp (T.K.); yujimizumizu0302@gmail.com (Y.M.); akaishi.mik.pz@yokohama-cu.ac.jp (M.A.); sakono@yokohama-cu.ac.jp (T.S.); akmeguro@yokohama-cu.ac.jp (A.M.); mizunobu@yokohama-cu.ac.jp (N.M.); 4 Department of Ophthalmology, Saiseikai Yokohamashi Nanbu Hospital, Yokohama 234-0054, Japan; yabukazu@gmail.com; 5 Department of Ophthalmology, Yokohama Sky Eye Clinic, Yokohama 220-0011, Japan; s.and.e.hata@gmail.com

**Keywords:** cataract surgery, dry eye disease, meibomian gland dysfunction, intense pulsed light, diquafosol, tear film, intraocular lens power calculation, refractive prediction error, tear breakup time, higher-order aberrations

## Abstract

**Objectives**: To compare the effects of two preoperative dry eye treatments—3% diquafosol sodium (DQS) and intense pulsed light with meibomian gland expression (IPL-MGX)—on refractive accuracy in cataract surgery and identify tear break-up time (TBUT) thresholds predictive of refractive success. **Methods**: In this prospective, paired-eye study, 62 patients (124 eyes) with meibomian gland dysfunction underwent bilateral cataract surgery with the same trifocal intraocular lens. One eye received DQS, while the fellow eye underwent four IPL-MGX sessions before biometry. Postoperative absolute prediction error (P-SE) was compared. TBUT and higher-order aberrations (HOAs) were evaluated. Logistic regression identified predictors of refractive accuracy, and receiver operating characteristic (ROC) analysis assessed the predictive value of TBUT for P-SE thresholds of <0.25 D and <0.50 D. **Results**: P-SE was significantly lower in IPL-MGX–treated eyes than in DQS-treated eyes (mean paired difference −0.11 D, *p* < 0.001). Success rates within <0.25 D and <0.50 D were higher with IPL-MGX (*p* < 0.01). TBUT and HOAs were predictors in univariate models, but only TBUT remained significant in the multivariable analysis (odds ratio, 4.90 per 1-s increase; 95% confidence interval, 1.92–12.51; *p* < 0.001). ROC analysis supported TBUT cutoffs of 7 s (<0.25 D) and 6 s (<0.50 D). **Conclusions**: IPL-MGX may improve refractive accuracy compared to DQS. TBUT appeared to be the most consistent predictor, and achieving ≥6 s was associated with higher likelihood of refractive success.

## 1. Introduction

Achieving accurate refractive outcomes is critical in modern cataract surgery, particularly with the widespread use of premium intraocular lenses (IOLs; multifocal and toric) that demand high precision [1]. Postoperative refractive error (difference between target and achieved refraction) greater than 1.0 diopter (D) is considered significant, and even smaller errors (±0.50 D or ±0.25 D) can impact patient satisfaction with multifocal IOLs [2,3]. Among various factors affecting IOL calculation accuracy, the ocular surface and tear film have emerged as important and modifiable contributors [1]. Errors in corneal measurements (keratometry) account for roughly 10% of refractive surprises after cataract surgery, often due to irregular or unstable corneal surface from dry eye disease (DED) [2]. Consequently, there is growing consensus that proactively treating DED, including meibomian gland dysfunction (MGD), prior to cataract surgery can improve refractive predictability and postoperative visual quality [1,4,5].

MGD, the leading cause of evaporative dry eye in older adults within the typical age range for cataract development is highly prevalent and compromises tear film stability. Lemp et al. reported MGD in approximately 86% of dry eye cases, underscoring its key role in ocular surface disturbance [6,7,8,9]. Multiple studies have validated this approach: pretreatment with topical lifitegrast (an anti-inflammatory dry eye medication), topical corticosteroids, or cyclosporine prior to biometry considerably improved the accuracy of IOL power predictions [1,2,4].

Topical 3% diquafosol sodium (DQS) is widely used for dry eye, particularly in Asia. As a P2Y_2_ receptor agonist, DQS stimulates aqueous and mucin secretion from conjunctival cells and may indirectly enhance lipid layer spread [10]. It increases tear volume, goblet cell density, and tear film stability, while reducing ocular surface staining [11]. Unlike artificial tears, DQS promotes endogenous tear and mucin production, targeting tear film instability at a biochemical level [10,11]. Clinical studies have shown its efficacy across dry eye subtypes, including MGD, with reported benefits on lipid layer thickness and tear break-up time (TBUT) [12,13]. In patients who have undergone cataract surgery, adjunctive DQS significantly improves tear film parameters; for example, a multicenter trial demonstrated increased TBUT (4.9 to 6.7 s, *p* < 0.001) and reduced corneal staining with preservative-free DQS [14,15]. Moreover, recent evidence suggests that DQS enhances preoperative keratometry reliability by improving ocular surface conditions and reducing astigmatism measurement variability [16].

Intense pulsed light with meibomian gland expression (IPL-MGX) is an office-based therapy that improves tear stability by reducing eyelid telangiectasia, inflammation, and liquefying meibum [17,18]. Clinical trials and meta-analyses demonstrate its remarkable benefits in TBUT, meibomian gland function, and dry eye symptoms [19,20]. Importantly, Kawagoe et al. demonstrated that preoperative IPL-MGX markedly enhanced refractive accuracy in cataract surgery, increasing the proportion of eyes within ±0.50 D of the target from approximately 55% to 93% (*p* < 0.01) [21]. These findings highlight the value of treating MGD before surgery.

This study aimed to compare the effects of two preoperative dry eye treatments—3% DQS and IPL-MGX—on postoperative refractive accuracy in cataract surgery as well as identify clinically meaningful predictors and thresholds, particularly TBUT, associated with refractive outcomes.

## 2. Materials and Methods

### 2.1. Study Design and Patients

This single-center, prospective, paired-eye comparative study included 124 eyes from 62 patients (28 males and 34 females; aged 50–80 years) scheduled for bilateral cataract surgery with implantation of a trifocal IOL (Clareon PanOptix, Alcon, Fort Worth, TX, USA). All patients were diagnosed with DED associated with MGD in both eyes, based on the Japanese Dry Eye Diagnostic Criteria—which include the presence of typical symptoms (e.g., ocular discomfort or visual disturbance) and a TBUT of <5 s [22]—as well as the MGD diagnostic criteria [23].

Dry eye symptoms were assessed using the validated Japanese version of the Ocular Surface Disease Index (OSDI) questionnaire [24]. Abnormalities around the meibomian gland orifices were identified when at least one of the following signs was observed: displacement of the anterior or posterior mucocutaneous junction, irregular lid margins, or dilated blood vessels. Obstruction was considered present when both orifice irregularities and reduced meibomian gland secretion were detected [23].

Key inclusion criteria encompassed: (1) bilateral cataracts suitable for diffractive trifocal IOL implantation, (2) patient preference for diffractive trifocal IOLs, (3) clinically significant MGD-related evaporative DED in both eyes, and (4) no history of prior dry eye treatment. Patients were excluded if they met any of the following criteria: (1) prior use of topical medications or punctal plugs for DED before enrollment; (2) coexisting skin conditions, blepharitis, or other dermatological disorders; (3) history of contact lens wear; (4) prior ocular surgery, ocular trauma, inflammation, corneal dystrophies, scarring, or any condition that could affect the ocular surface; (5) diagnosis of diabetes mellitus; or (6) selection of alternative preoperative DED therapies after enrollment.

All participants provided written informed consent. The study adhered to the tenets of the Declaration of Helsinki and was approved by the Ethics Committee of Tsurumi Chuo Eye Clinic (approval number: 2024-002; approval date: 3 April 2024).

### 2.2. Interventions

Assignment of IPL-MGX or 3% DQS to the right or left eye was carried out using a permuted block randomization method with a block size of 4. The randomization sequence was generated by a study coordinator who was not involved in outcome assessment, and allocation was concealed until assignment.

In each patient, one eye was allocated to receive IPL-MGX and the fellow eye to receive 3% DQS over the same preoperative period. No additional dry eye medications or procedures were permitted during the intervention phase. The specific treatment protocols for IPL-MGX and DQS are described in the following section.

At each treatment visit, the ophthalmologist monitored the IPL-treated eye for potential adverse events, including erythema, edema, blister formation, and scarring, and documented any findings.

#### Preoperative Interventions

**Figure 1:**
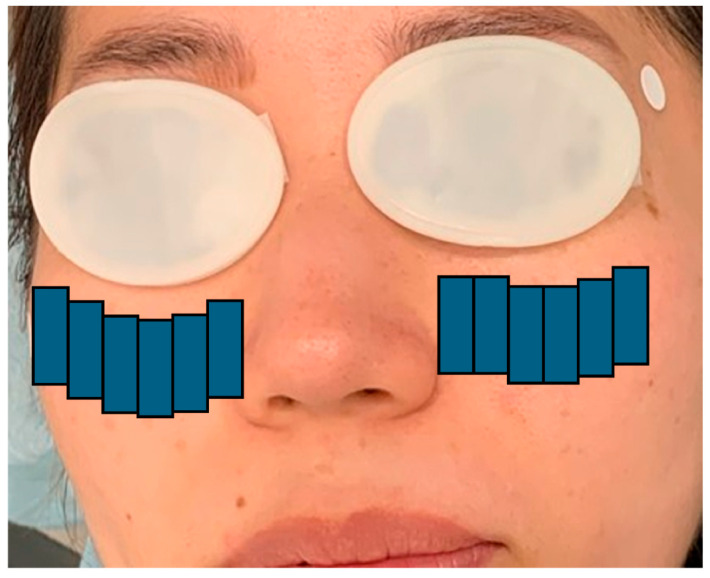
Intense pulsed light treatment protocol—Step 1: Twelve pulses were delivered to the infraorbital and lower eyelid area using a double-pass technique with a 15 × 35 mm guide light. The schematic shows the procedure with eye patches for demonstration only and does not portray a real patient [25].

**Figure 2:**
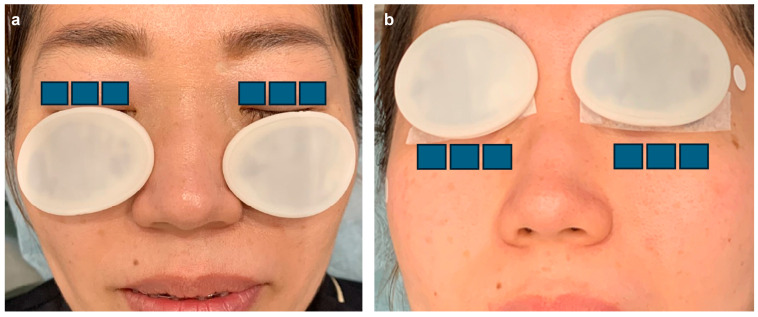
Intense pulsed light treatment protocol—Step 2: Three pulses were applied to the upper (**a**) and lower (**b**) eyelids using a single-pass technique with an 8 × 15 mm guide light. The illustration depicts the use of eye patches for demonstration purposes and is not an actual patient representation [25].

In the IPL-MGX group, treatment was performed using a commercially available IPL system (M22; Lumenis, Yokne’am Illit, Israel) in triple-pulse mode, followed by manual meibomian gland expression approximately 10 min later. Standard eye protection was used throughout the procedure. Treatment parameters and protocols were consistent with those described in prior reports (Figure 1 and Figure 2) [25].

In the DQS group, preservative-free 3% diquafosol ophthalmic solution (Diquas^®^-S; Santen Pharmaceutical, Osaka, Japan) was instilled six times daily from the initiation of the first IPL session until preoperative biometry, corresponding to a 6–8-week treatment period. Compliance was assessed through patient interviews and verification of returned used vials.

The 3% DQS treatment duration (6–8 weeks) was selected to reflect the typical preoperative dry eye management window in cataract surgery, during which biometry must be completed.

All eyes received a standardized perioperative regimen of topical antibiotics, steroids, and nonsteroidal anti-inflammatory drugs, according to established clinical practice. No patients continued DQS or IPL after surgery.

### 2.3. Cataract Surgery and IOL Selection

Approximately 5 days (mean 5.0 ± 2.1 days) after the preoperative examination, patients underwent cataract surgery in both eyes. Surgeries were performed by a single experienced surgeon (T.T.) at Tsurumi Chuo Eye Clinic, Yokohama, Japan. Both eyes were typically operated on within a one-week interval. Standard phacoemulsification with femtosecond laser-assisted steps (LenSx; Alcon Laboratories, Inc., Aliso Viejo, CA, USA) was performed in all cases, and a Clareon PanOptix trifocal IOL (Alcon Laboratories, Inc.) was implanted in the capsular bag of each eye. The targeted refraction for both eyes was emmetropia (plano).

IOL power calculation for each eye was based on that eye’s preoperative biometry, primarily using the Barrett Universal II formula [26]. Because preoperative interventions could influence keratometry, IOL power occasionally differed between the two eyes.

This study followed an open-label design, as neither the surgeon nor the patients were masked to treatment allocation. Masking was not feasible because the two interventions (IPL-MGX versus topical DQS) differed substantially in their mode of administration and patient experience, making it impossible to conceal allocation. However, key outcomes such as keratometry, refraction, and IOL power calculation were based on standardized, objective measurements, thereby minimizing the potential influence of observer bias. Apart from the assigned preoperative dry eye treatment, pre- and postoperative care and surgical procedures were identical in both eyes.

### 2.4. Evaluation of Ocular Surface Status, Biometric Parameters, and Predictive Accuracy of Postoperative Refraction

Tear film stability was evaluated using TBUT [27,28], and corneal higher-order aberrations (HOAs) were assessed as indicators of ocular surface quality [29,30]. Key biometric parameters relevant to IOL power calculation—including axial length (AL), anterior chamber depth (ACD), and mean keratometry (mean-K)—were obtained [31,32]. The accuracy of predicted postoperative spherical equivalent (P-SE) was determined as the absolute difference between the predicted value (using the Barrett Universal II formula with the IOLMaster 700, Carl Zeiss Meditec AG, Jena, Germany) and the subjective postoperative spherical equivalent (S-SE).

All measurements were performed at baseline and again approximately one week after completing the four-session preoperative treatment (IPL-MGX or 3% DQS). Cataract surgery was then typically performed approximately one week later.

For TBUT assessment, fluorescein was instilled into the inferior conjunctival sac, and the average of two measurements was recorded [33]. HOAs within the central 4 mm were measured using anterior segment optical coherence tomography (CASIA 2; Tomey, Nagoya, Japan) with Zernike polynomial analysis, and the total magnitude of third- to sixth-order aberrations was expressed as the root mean square [34]. Biometric parameters were measured with the IOLMaster 700. Subjective refraction was obtained one month postoperatively by a certified examiner using an ETDRS chart, and the absolute difference between P-SE and S-SE was taken as the measure of refractive accuracy.

### 2.5. Categorical Accuracy Outcomes

We evaluated the proportion of eyes achieving refractive outcomes within commonly reported error thresholds in cataract surgery: ≤0.25 D, ≤0.50 D, ≤0.75 D, and ≤1.00 D of the target spherical equivalent. For each threshold, the refractive outcome of each eye was classified as a “success” (within the specified threshold) or a “failure” (exceeding the threshold). Because each patient contributed one eye to the IPL-MGX group and the fellow eye to the DQS group, success rates could be compared in a paired manner.

### 2.6. Logistic Regression and Cutoff Analysis for Predicting Refractive Accuracy

To assess predictors of refractive accuracy, logistic regression analyses were performed. Univariate models first evaluated whether post-treatment TBUT and HOAs individually influenced the likelihood of achieving accurate postoperative refraction. Multivariate models were then used to determine whether these variables remained independent predictors after adjustment for potential confounders.

For exploratory analyses, receiver operating characteristic (ROC) curves were generated to identify clinically relevant cutoff values of TBUT and HOAs for predicting successful refractive outcomes, defined as absolute prediction errors of <0.25 D and <0.50 D. Sensitivity, specificity, and area under the curve (AUC) were calculated, and the optimal cutoff was determined using the Youden Index. The aim was to establish actionable TBUT thresholds that could inform surgical planning.

### 2.7. Statistical Analysis

All analyses were performed using SPSS software, version 29.0 (IBM Corp., Armonk, NY, USA). A *p*-value < 0.05 was considered statistically significant.

This study included 62 patients (124 eyes). The required sample size was calculated a priori using G*Power (version 3.1.9.7; Universität Düsseldorf, Germany) for a paired-eye design. Assuming an effect size of 0.5, α = 0.05, and power = 0.80, the minimum sample size was estimated at 27 patients (54 eyes). The final enrollment exceeded this requirement, ensuring adequate statistical power.

Comparisons between IPL-MGX- and 3% DQS-treated eyes were performed on a paired basis. For continuous variables (absolute prediction error, TBUT, HOAs), normality was tested using the Shapiro–Wilk test [35]. Non-normally distributed data were analyzed using the Wilcoxon signed-rank test [36].

Categorical outcomes (proportion of eyes within ±0.25 D, ±0.50 D, ±0.75 D, and ±1.00 D of target refraction) were compared using McNemar’s test for paired nominal data [37,38].

Correlations between ocular surface metrics (TBUT, HOAs) and refractive accuracy were assessed using Spearman’s rank correlation coefficient [39], and 95% confidence intervals (CIs) were estimated by percentile bootstrap with 2000 replicates.

Logistic regression was performed to identify predictors of refractive success (<0.50 D vs. ≥0.50 D) [40]. TBUT (per 1-s increase) and HOAs (rescaled per 0.01 µm) were included as explanatory variables. Univariate analyses used cluster-robust standard errors, and multivariable models were fitted using generalized estimating equations (GEE) with an exchangeable correlation structure to account for within-patient clustering [40]. Odds ratios (ORs) with 95% CIs were reported [41]. Model fit was evaluated using the model chi-square test and Nagelkerke R^2^ statistic [42].

For exploratory analysis, ROC curves were generated to determine clinically relevant TBUT cutoffs for predicting refractive accuracy (<0.25 D and <0.50 D). The AUC was calculated with 95% CIs estimated using a 2000-replicate bootstrap, and sensitivity and specificity were accompanied by Wilson 95% CIs. Optimal thresholds were identified using the Youden Index [34]. Additional cutoff candidates (5, 6, 7, and 8 s) were also tested, and differences in success rates above versus below each threshold were assessed with chi-square tests [43].

Continuous variables are presented as mean ± standard deviation (SD) or median with interquartile range (IQR), and categorical variables as counts and percentages.

We performed an additional subgroup analysis by age and sex to determine if the IPL–MGX treatment effects on prediction error differed by these factors.

Additionally, we conducted within-group comparisons of pre- versus post-treatment values for TBUT, HOAs, and mean keratometry (average K) in each treatment arm (3% DQS and IPL-MGX). Paired t-tests or Wilcoxon signed-rank tests were used as appropriate based on data normality. These analyses aimed to assess the intraocular effects of each dry eye therapy prior to cataract surgery.

## 3. Results

### 3.1. Baseline Patient Characteristics

A total of 62 patients (124 eyes; 28 male and 34 female; aged 50–80 years) with MGD-related dry eye were enrolled. All patients completed the assigned preoperative treatment, underwent cataract surgery in both eyes, and were included in the final analysis. No participants dropped out, and no eyes were excluded due to missing data. Nursing staff confirmed high adherence to the assigned treatment in all participants.

No IPL-associated adverse events (such as erythema, edema, blistering, or scarring) or DQS-related adverse effects were observed. At baseline, ocular biometric parameters—including AL, ACD, mean-K, TBUT, and corneal HOAs—did not differ significantly between paired eyes. Baseline comparability is summarized in Table 1, and subsequent results are reported on a paired-eye basis.

### 3.2. Post-Treatment Change in Keratometric Values

Post-treatment mean-K values are summarized in Table 2. A statistically significant paired difference was observed: the IPL-MGX–treated eyes showed a lower mean-K value (43.21 ± 1.85 D) compared with fellow eyes treated with 3% DQS (43.35 ± 1.76 D; mean paired difference = −0.14 D, 95% CI: −0.24 to −0.04; *p* = 0.013). This finding indicates that IPL-MGX contributed to a modest but statistically significant reduction in preoperative keratometric values.

### 3.3. Tear Film Stability and Ocular Surface Metrics

Post-treatment tear film and optical quality metrics are summarized in Table 3. Compared with fellow eyes treated with 3% DQS, IPL-MGX–treated eyes showed significantly longer TBUT (6.70 ± 1.27 vs. 5.72 ± 1.11 s; mean paired difference = +0.98 s, 95% CI: +0.66 to +1.30; *p* < 0.001) and lower HOAs (0.264 ± 0.028 vs. 0.287 ± 0.025 µm; mean paired difference = −0.022 µm, 95% CI: −0.030 to −0.015; *p* < 0.001). These results indicate superior improvement in ocular surface stability and optical quality following IPL-MGX.

Within-group comparisons revealed statistically significant improvements in TBUT and HOAs from baseline to post-treatment in both groups. In the 3% DQS group, TBUT increased from 3.92 ± 0.86 s to 5.72 ± 1.11 s (*p* < 0.001) and HOAs decreased from 0.340 ± 0.026 µm to 0.287 ± 0.025 µm (*p* < 0.001). In the IPL-MGX group, TBUT increased from 3.89 ± 0.83 s to 6.70 ± 1.27 s (*p* < 0.001) and HOAs decreased from 0.340 ± 0.027 µm to 0.264 ± 0.028 µm (*p* < 0.001). Average K showed no significant change in the 3% DQS group (43.56 ± 1.58 D to 43.35 ± 1.76 D, *p* = 0.27), whereas the IPL-MGX group demonstrated a modest but significant decrease (43.60 ± 1.45 D to 43.21 ± 1.85 D, *p* = 0.03).

### 3.4. Refractive Accuracy

P-SE findings are summarized in Table 4. Eyes treated with IPL-MGX demonstrated significantly lower absolute prediction error (0.28 ± 0.14 D) compared with fellow eyes treated with 3% DQS (0.40 ± 0.18 D; mean paired difference = −0.11 D, 95% CI: −0.14 to −0.09; *p* < 0.001). This finding indicates that IPL-MGX was associated with superior postoperative refractive precision.

We stratified the primary outcome (postoperative refractive prediction error) by age and sex to evaluate subgroup differences. In both the 3% DQS and IPL-MGX groups, there were no significant differences in prediction error between younger (<75 years) and older patients (≥75 years) or between male and female patients. For example, in the 3% DQS group, younger patients had a mean prediction error of 0.37 ± 0.19 D vs. 0.42 ± 0.18 D in older patients (*p* = 0.15, Mann–Whitney U test). Similarly, in the IPL-MGX group, the mean error was 0.26 ± 0.13 D in younger patients vs. 0.29 ± 0.15 D in older patients (*p* = 0.34). Prediction error was also comparable between sexes: in the 3% DQS arm, males vs. females had 0.38 ± 0.17 D vs. 0.41 ± 0.20 D (*p* = 0.60), whereas in the IPL-MGX arm, 0.26 ± 0.10 D vs. 0.29 ± 0.18 D, respectively (*p* = 0.88). These analyses indicate that neither age nor sex significantly influenced refractive prediction accuracy within each treatment group.

### 3.5. Categorical Postoperative Refractive Accuracy

**Figure 3:**
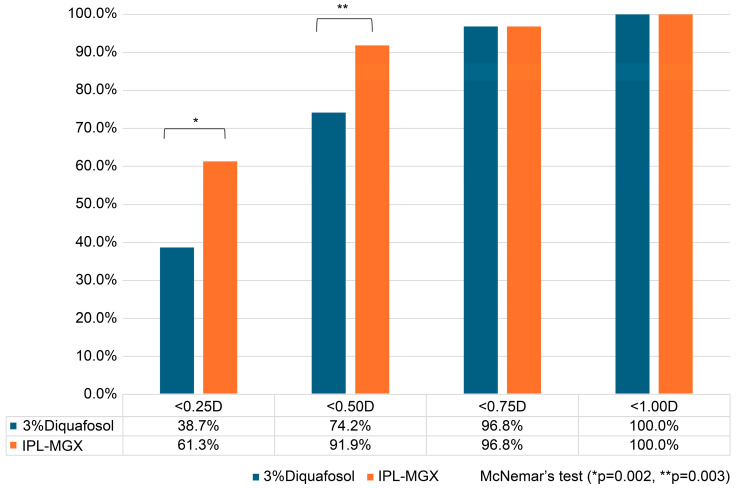
Proportion of eyes achieving postoperative refractive accuracy at different thresholds (<0.25 D, <0.50 D, <0.75 D, and <1.00 D) in paired-eye comparisons of IPL-MGX versus 3% Diquafosol. Statistical comparisons were performed using McNemar’s test (*p* = 0.002 for <0.25 D; *p* = 0.003 for <0.50 D; not significant at higher thresholds). IPL-MGX, intense pulsed light with meibomian gland expression.

The proportions of eyes achieving different levels of absolute postoperative refractive error are summarized in Figure 3. At the <0.25 D threshold, 61.3% of IPL-MGX–treated eyes achieved this accuracy compared with 38.7% of fellow eyes treated with 3% DQS. At the <0.50 D threshold, 91.9% of IPL-MGX-treated eyes were within this range compared with 74.2% of DQS-treated eyes. Paired-eye analysis using McNemar’s test confirmed that these differences were statistically significant (<0.25 D: discordant pairs = 2 vs. 15, paired difference = +21.3%, 95% CI: +8.1% to +34.6%, *p* = 0.002; <0.50 D: discordant pairs = 1 vs. 12, paired difference = +18.0%, 95% CI: +6.4% to +29.6%, *p* = 0.003).

At higher thresholds, the differences were no longer significant, as both treatments achieved comparable accuracy (<0.75 D: 96.8% vs. 96.8%, *p* = 1.000; <1.0 D: 100% vs. 100%, *p* = 1.000). These findings indicate that IPL-MGX treatment was associated with a significantly greater likelihood of achieving high refractive precision, particularly within ±0.25 D and ±0.50 D of the intended correction.

### 3.6. Correlation Between Ocular Surface Parameters and Postoperative Refractive Error

The results of correlation analyses are summarized in Table 5. In group-wise analyses, post-treatment TBUT was negatively correlated with postoperative absolute refractive error in both groups (DQS: ρ = −0.665, 95% CI −0.793 to −0.491, *p* < 0.001; IPL: ρ = −0.621, 95% CI −0.777 to −0.414, *p* < 0.001). Conversely, corneal HOAs showed positive correlations with refractive error (DQS: ρ = 0.537, 95% CI 0.303 to 0.728, *p* < 0.001; IPL: ρ = 0.734, 95% CI 0.578 to 0.834, *p* < 0.001). Bootstrap-based 95% CIs were estimated using 2000 replicates.

### 3.7. Predictive Analyses of Refractive Accuracy

#### 3.7.1. Univariate Logistic Regression

As shown in Table 6, longer post-treatment TBUT was significantly associated with higher odds of achieving refractive accuracy < 0.50 D (OR 3.77 per 1-s increase, 95% CI 1.94–7.33, *p* < 0.001), whereas higher HOAs were associated with lower odds (OR 0.64 per 0.01-µm increase, 95% CI 0.49–0.83, *p* < 0.001).

#### 3.7.2. Multivariable Analysis Accounting for Paired Eyes

In the GEE model accounting for inter-eye clustering (Table 7), TBUT remained an independent predictor of refractive accuracy < 0.50 D (OR 4.90 per 1-s increase, 95% CI 1.92–12.51, *p* < 0.001). In contrast, HOAs were not significantly associated after adjustment (OR 1.12 per 0.01-µm increase, 95% CI 0.80–1.57, *p* = 0.506).

#### 3.7.3. ROC Analysis and Clinical Thresholds

ROC analysis confirmed the predictive value of TBUT, with an AUC of 0.826 (95% CI 0.733–0.907; bootstrap, 2000 replicates). As summarized in Table 8, a cutoff of 6 s provided a balanced profile (sensitivity 0.713 [95% CI 0.618–0.792], specificity 0.667 [95% CI 0.454–0.828]), while 7 s yielded very high specificity but reduced sensitivity (0.545 [95% CI 0.448–0.638], specificity 0.952 [95% CI 0.773–0.992]). These findings support TBUT ≥6 s as a practical presurgical benchmark, with ≥7 s representing a stricter criterion.

#### 3.7.4. Supplementary Findings on TBUT Distribution

Post-treatment TBUT distribution further supported the predictive role of tear film stability. A significantly larger proportion of IPL-MGX–treated eyes achieved TBUT ≥ 6 s compared with fellow eyes treated with DQS (79.0% [49/62] vs. 50.0% [31/62], *p* = 0.001). Moreover, among eyes achieving a postoperative refractive error < 0.50 D, 70.9% (73/103) had TBUT ≥ 6 s, whereas only 33.3% (7/21) of eyes with ≥0.50 D error reached this level (*p* = 0.002). These findings suggest that attaining the TBUT threshold of 6 s is closely linked to refractive accuracy and is more consistently achieved following IPL-MGX treatment (Table 9).

## 4. Discussion

### 4.1. Main Findings and Interpretation

This paired-eye study involving 62 patients (124 eyes) provides robust evidence that preoperative management of MGD-related dry eye with IPL-MGX considerably improves refractive accuracy following cataract surgery compared with 3% DQS. Since this study followed a within-subject design, each patient served as their own control, thereby minimizing interindividual variability and strengthening the validity of the observed treatment effect [36].

At the patient level, paired-eye analysis confirmed that IPL-MGX yielded superior refractive outcomes. The absolute prediction error was significantly lower in IPL-MGX–treated eyes (0.28 ± 0.14 D) than in fellow eyes treated with DQS (0.40 ± 0.18 D), with a mean paired difference of −0.11 D (95% CI: −0.14 to −0.09; *p* < 0.001). Moreover, categorical accuracy analyses demonstrated that IPL-MGX eyes were significantly more likely to achieve high precision: 61.3% of IPL-MGX eyes versus 38.7% of DQS eyes achieved < 0.25 D error (discordant pairs 2 vs. 15, paired difference +21.3%, 95% CI: +8.1% to +34.6%, *p* = 0.002) and 91.9% versus 74.2% achieved < 0.50 D error (discordant pairs 1 vs. 12, paired difference +18.0%, 95% CI: +6.4% to +29.6%, *p* = 0.003). At higher thresholds (<0.75 D and <1.0 D), both treatments performed comparably. These findings highlight that the advantage of IPL-MGX lies particularly in achieving the highest levels of refractive precision. Such differences, although numerically modest, are clinically meaningful in the context of diffractive trifocal IOLs, where even a 0.25–0.50 D deviation may substantially compromise image quality and patient satisfaction [1,5].

Mechanistically, our results support the concept that ocular surface optimization with IPL-MGX enhances the reliability of biometric measurements by stabilizing the tear film and improving optical quality. IPL-MGX–treated eyes demonstrated significantly greater improvement in TBUT (mean difference +0.98 s, *p* < 0.001) and reduction in corneal HOAs (mean difference −0.022 µm, *p* < 0.001) compared with DQS. Post-treatment TBUT showed a strong negative correlation with refractive error, and logistic regression identified TBUT as an independent predictor of achieving <0.50 D accuracy (multivariable OR 4.90 per 1-s increase, 95% CI 1.92–12.51, *p* < 0.001). ROC analysis further indicated that a TBUT threshold of ≥6 s provided a practical balance of sensitivity and specificity [29,33], and eyes treated with IPL-MGX were significantly more likely to reach this benchmark (79.0% vs. 50.0%, *p* = 0.001). These data suggest that tear film stability—more consistently achieved with IPL-MGX—serves as a key determinant of refractive precision.

The within-group analysis further confirmed that both treatments produced statistically significant improvements in tear film stability and optical quality prior to surgery. Although both therapies led to enhanced TBUT and reduced HOAs, the magnitude of improvement was consistently greater with IPL-MGX. The modest but statistically significant reduction in average K observed only in the IPL-MGX group may reflect improved tear film regularity and reduced surface distortion, which could contribute to better IOL calculation accuracy. These findings support the role of ocular surface optimization not only in enhancing absolute refractive precision but also in improving the consistency of biometric measurements.

### 4.2. Comparison with Existing Literature

Our findings are consistent with those of Kawagoe et al. [21], who reported that IPL-MGX improved refractive predictability prior to trifocal IOL implantation. In their prospective study, the proportion of eyes within ±0.25 D increased markedly after IPL treatment. Our study extends this evidence by employing a paired-eye design in 62 patients (124 eyes), thereby controlling for interindividual variability and demonstrating within-subject superiority of IPL-MGX at both the ±0.25 D and ±0.50 D thresholds. Similarly, Biela et al. [2] reported that IPL improved TBUT and refractive outcomes in patients with dry eye, although their non-paired design limited the precision of causal inference. Together, these findings reinforce that ocular surface optimization with IPL confers measurable benefits in refractive accuracy.

In contrast, while 3% DQS has proven efficacy in stimulating mucin and aqueous secretion [17,20,21], its effect appears limited in MGD-dominant evaporative dry eye, where lipid deficiency and tear film instability are the primary drivers [5]. In our cohort, DQS improved TBUT from 3.88 s to 5.45 s; however, this improvement remained inferior to that observed in the IPL group (6.70 s), and HOAs were not significantly reduced. Multivariable analysis further confirmed that TBUT—but not HOAs—was an independent predictor of achieving refractive accuracy < 0.50 D (OR 4.90 per 1-s increase, 95% CI 1.92–12.51, *p* < 0.001). This is consistent with prior work indicating that HOAs are strongly associated with ocular surface irregularity and vision-related quality of life in dry eye [30,34] but may not independently predict refractive precision once tear stability is considered. These results suggest that TBUT serves as a more comprehensive biomarker of ocular surface integrity, encompassing both tear stability and optical quality, which is consistent with prior reports on short-TBUT–type dry eye [29,33].

Although longer-term 3% DQS therapy has been reported to enhance lipid secretion in MGD, extending treatment to such durations is not feasible in routine preoperative cataract workflows. Therefore, the present study adopted a clinically realistic treatment window.

### 4.3. Clinical Thresholds for Tear Stability

One of the most clinically novel contributions of this study is the identification of specific TBUT thresholds that predict postoperative refractive accuracy with high reliability. To our knowledge, this is the first clinical investigation to systematically propose and validate practical TBUT cutoffs that can serve as preoperative decision-making tools in cataract surgery. ROC curve analysis revealed that a TBUT of ≥6 s provided the most balanced predictive profile for refractive error <0.50 D, with an AUC of 0.826 (95% CI: 0.733–0.907), sensitivity of 0.713, and specificity of 0.667. A stricter cutoff of ≥7 s achieved very high specificity (0.952) but at the expense of reduced sensitivity (0.545). These findings suggest that achieving a TBUT of at least 6 s is a practical, evidence-based preoperative benchmark, whereas 7 s may serve as a more conservative criterion in selected cases.

Earlier studies have linked TBUT < 5 s with visual fluctuations, compromised optical quality, and keratometry repeatability issues [39,44]. Our study extends these observations by establishing outcome-driven thresholds that are directly tied to refractive precision. This transition from descriptive tear film assessment to predictive, quantifiable benchmarks represents an important step forward in preoperative planning for premium IOL surgery. Notably, these thresholds are also in line with prior concepts of short-TBUT–type dry eye [29,33], thereby reinforcing their clinical plausibility.

Interestingly, although HOAs were correlated with refractive accuracy in the univariate analysis, they did not retain significance in the multivariable model. This supports the interpretation that TBUT acts as a broader physiological indicator of ocular surface integrity, encompassing both functional and optical stability, whereas HOAs may represent a downstream manifestation of instability rather than an independent, modifiable predictor [30,34].

### 4.4. Clinical Implications

With the growing prevalence of premium IOL implantation, particularly trifocal and extended depth of focus designs, optimizing refractive outcomes has become indispensable. Even small residual errors (±0.25–0.50 D) can induce dysphotopsia, reduce spectacle independence, and diminish patient satisfaction [1,8]. Our findings demonstrate that preoperative IPL-MGX considerably increased the likelihood of achieving refractive accuracy within these critical thresholds and that eyes reaching a TBUT ≥ 6 s were far more likely to achieve < 0.50 D error. These results provide clinicians with a practical and evidence-based preoperative benchmark for surgical planning.

For clinicians without access to IPL, 3% DQS remains a reasonable alternative for milder cases of dry eye, particularly those with predominantly aqueous or mucin deficiency [17,20,21]. However, its capacity to stabilize the tear film and smooth the optical surface was limited in our study, particularly in patients with MGD-dominant evaporative dry eye. In contrast, IPL-MGX demonstrated superior improvements in TBUT and refractive accuracy in our paired-eye trial, underscoring its value as an effective intervention for MGD-related dry eye. Although our study did not grade MGD severity, prior reports have highlighted the greatest benefits of IPL therapy in moderate to severe MGD [8,9,10,11], which suggests that the impact observed here may be especially relevant in those populations.

Importantly, IPL therapy was well tolerated in our cohort, with no treatment-related adverse events observed. This aligns with prior prospective and randomized clinical trials [10,11,12,13], further confirming its safety and reproducibility. Given its non-invasive nature, strong safety profile, and demonstrable impact on refractive precision, broader adoption of IPL-MGX as part of routine preoperative dry eye optimization appears justified, particularly in candidates undergoing premium IOL surgery.

The absence of any significant age- or sex-related differences in prediction error suggests that both treatments maintained refractive accuracy across these demographic subgroups. In particular, the efficacy of IPL-MGX was consistent among younger vs. older patients and across both sexes. Our results indicate that patients ≥ 75 years achieved similar refractive outcomes as those < 75 years and that female patients fared as well as males in terms of predictive accuracy. One explanation is that optimizing the ocular surface with 3% DQS or IPL-MGX might have mitigated age- or sex-related differences in biometry. We found no meaningful disparity in the magnitude of prediction error between sexes. Taken together, these findings support the broad applicability of both 3% DQS and IPL-MGX interventions: they can be effectively utilized to improve refractive outcomes regardless of patient age or sex, without the need for demographic-specific adjustments.

### 4.5. Limitations

While this study provides valuable insights, some limitations should be acknowledged. First, although the paired-eye design reduces intersubject variability, it does not eliminate interocular differences in tear physiology and ocular surface status, which may still influence outcomes. Second, the study followed an open-label design; masking was not feasible due to the distinct nature of the interventions, and observer bias cannot be fully excluded despite reliance on standardized, objective measurements. Third, while our analytic plan incorporated methods to address clustering and precision (e.g., paired tests, cluster-robust standard errors, generalized estimating equations, and bootstrap-based confidence intervals), residual within-patient correlation and model specification could still lead to overly narrow CIs or seemingly large effect estimates; therefore, predictive findings should be interpreted with caution and validated independently. Fourth, follow-up was limited to the early postoperative period (1 month), so the durability of both tear film improvements and refractive precision remains uncertain. Additionally, postoperative TBUT and HOAs were not collected because these parameters are substantially influenced by postoperative medications and inflammation and therefore do not reliably reflect the effect of preoperative dry eye treatment. Fifth, we focused on refractive accuracy and objective ocular surface metrics; subjective visual quality, contrast sensitivity, and patient-reported outcomes were not assessed. Sixth, this was a single-center, single-surgeon study, which may limit generalizability. Seventh, although longer-term 3% DQS therapy (≥3 months) has been reported to enhance lipid secretion in MGD [45], such extended regimens are not routinely feasible in preoperative cataract workflows. The present study therefore focused on clinically realistic treatment durations. Finally, the exploratory threshold analysis (e.g., TBUT ≥ 6 s and ≥ 7 s) entailed multiple comparisons; although we used the Youden Index and reported CIs, these cutoffs should be regarded as hypothesis-generating pending external validation.

### 4.6. Future Directions

Future research should aim to validate these findings in larger, multicenter, randomized controlled trials with longer follow-up. Such studies could confirm the durability of IPL-MGX–related improvements in tear film stability and refractive precision, while accounting for inter-surgeon and inter-center variability. Additionally, more advanced statistical modeling—such as mixed-effects or penalized logistic regression—may help refine predictive accuracy and further address clustering and overfitting concerns.

Additionally, examining whether adjunctive therapies (e.g., lifitegrast, topical corticosteroids) or sequential treatment strategies can enhance outcomes when combined with IPL-MGX, particularly in eyes with suboptimal TBUT improvement, would prove valuable. Furthermore, investigating whether early IPL intervention reduces the need for intraocular lens exchange or postoperative refractive enhancement in patients with premium IOLs could have important clinical implications.

Moreover, future studies may explore combination or sequential regimens—such as initiating treatment with IPL-MGX followed by maintenance with topical DQS—to enhance both tear film stability and meibomian gland function. These multimodal strategies could potentially produce additive or synergistic effects, particularly in patients with moderate to severe MGD. Controlled trials evaluating such protocols may help establish more effective and individualized preoperative dry eye management pathways.

Finally, integration of advanced ocular surface imaging and optical metrics—such as dynamic wavefront analysis, tear interferometry, and corneal topography dynamics—may provide deeper insights into the relationship between tear film stability, optical quality, and refractive predictability in this patient population.

## 5. Conclusions

In this paired-eye, prospective study of 62 patients (124 eyes), preoperative ocular surface optimization with IPL-MGX remarkably improved refractive accuracy in cataract surgery compared with 3% DQS. TBUT emerged as the most powerful and independent predictor of refractive precision, and specific TBUT thresholds—particularly ≥6 s—were identified as practical, evidence-based benchmarks for surgical readiness. These findings establish a new, objective framework for preoperative planning, highlighting TBUT as a modifiable biomarker that links ocular surface stability to refractive predictability and visual outcomes.

## Figures and Tables

**Table 1:** Baseline ocular characteristics of paired eyes (n = 62 patients, 124 eyes).

Parameter	3% Diquafosol (Mean ± SD)	IPL-MGX (Mean ± SD)	*p*-Value
AL (mm)	23.71 ± 1.37	23.69 ± 1.34	0.377
ACD (mm)	2.94 ± 0.36	2.95 ± 0.35	0.399
Mean-K (D)	43.56 ± 1.58	43.60 ± 1.45	0.178
TBUT (s)	3.92 ± 0.86	3.89 ± 0.83	0.718
HOAs (μm)	0.34 ± 0.03	0.34 ± 0.03	0.840

All values are presented as mean ± standard deviation. Comparisons were performed using paired-eye analysis. All 62 patients (124 eyes) completed the study with no dropouts or missing data. AL, axial length; ACD, anterior chamber depth; Mean-K, mean keratometry; TBUT, tear break-up time; HOAs, higher-order aberrations.

**Table 2:** Post-treatment average keratometry (K) values in paired eyes (n = 62 patients, 124 eyes).

Parameter	3% Diquafosol (Mean ± SD)	IPL-MGX (Mean ± SD)	Mean Paired Difference (95% CI)	*p*-Value
Mean-K (D)	43.35 ± 1.76	43.21 ± 1.85	−0.14 (−0.24 to −0.04)	0.013

Values represent paired comparisons between fellow eyes. *p*-values are derived from Wilcoxon signed-rank test. Mean-K, mean keratometry; IPL-MGX, intense pulsed light with meibomian gland expression; SD, standard deviation; CI, confidence interval.

**Table 3:** Post-treatment tear film stability and ocular surface metrics in paired eyes (n = 62 patients, 124 eyes).

Parameter	3% Diquafosol (Mean ± SD)	IPL-MGX (Mean ± SD)	Mean Paired Difference (95% CI)	*p*-Value
TBUT (s)	5.72 ± 1.11	6.70 ± 1.27	+0.98 (+0.66 to +1.30)	<0.001
HOAs (μm)	0.287 ± 0.025	0.264 ± 0.028	−0.022 (−0.030 to −0.015)	<0.001

Values represent paired comparisons between fellow eyes. *p*-values are derived from Wilcoxon signed-rank test. TBUT, tear break-up time; HOAs, higher-order aberrations; IPL-MGX, intense pulsed light with meibomian gland expression; SD, standard deviation; CI, confidence interval.

**Table 4:** Postoperative absolute prediction error (P-SE) in paired eyes (n = 62 patients, 124 eyes).

Parameter	3% Diquafosol (Mean ± SD)	IPL-MGX (Mean ± SD)	Mean Paired Difference (95% CI)	*p*-Value
Absolute prediction error (D)	0.40 ± 0.18	0.28 ± 0.14	−0.11 (−0.14 to −0.09)	<0.001

Values represent paired comparisons between fellow eyes. *p*-values are derived from Wilcoxon signed-rank test. IPL-MGX, intense pulsed light with meibomian gland expression; CI, confidence interval; SD, standard deviation.

**Table 5:** Correlation between ocular surface parameters and postoperative refractive error (n = 62 pairs).

Group	Parameter	ρ (Spearman)	95% CI	*p*-Value
3% Diquafosol	TBUT vs. Refractive error	−0.665	−0.793 to −0.491	<0.001
	HOAs vs. Refractive error	0.537	0.303 to 0.728	<0.001
IPL-MGX	TBUT vs. Refractive error	−0.621	−0.777 to −0.414	<0.001
	HOAs vs. Refractive error	0.734	0.578 to 0.834	<0.001

ρ = Spearman’s rank correlation coefficient. 95% CIs are estimated using 2000-replicate percentile bootstrap. TBUT, tear break-up time; IPL-MGX, intense pulsed light with meibomian gland expression; HOAs, higher-order aberrations; CI, confidence interval.

**Table 6:** Univariate logistic regression for predictors of refractive accuracy < 0.50 D (n = 62 patients, 124 eyes).

Predictor	OR (95% CI)	*p*-Value
TBUT (per 1 s)	3.77 (1.94–7.33)	<0.001
HOAs (per 0.01 μm)	0.64 (0.49–0.83)	<0.001

TBUT, tear break-up time; HOAs, higher-order aberrations; OR, odds ratio; CI, confidence interval.

**Table 7:** Multivariable GEE logistic regression accounting for paired eyes (success = refractive error < 0.50 D).

Predictor	OR (95% CI)	*p*-Value
TBUT (per 1 s)	4.90 (1.92–12.51)	<0.001
HOAs (per 0.01 μm)	1.12 (0.80–1.57)	0.506

TBUT, tear break-up time; HOAs, higher-order aberrations; OR, odds ratio; CI, confidence interval; GEE, generalized estimating equation.

**Table 8:** ROC analysis of TBUT for predicting refractive accuracy < 0.50 D.

Threshold	Sensitivity (95% CI)	Specificity (95% CI)
≥6 s	0.713 (0.618–0.792)	0.667 (0.454–0.828)
≥7 s	0.545 (0.448–0.638)	0.952 (0.773–0.992)

ROC, receiver operating characteristic; TBUT, tear break-up time; CI, confidence interval.

**Table 9:** Proportion of eyes with TBUT ≥ 6 s by treatment group and refractive accuracy.

Comparison	TBUT ≥ 6 s	TBUT < 6 s	% ≥6 s	*p*-Value
IPL-MGX (n = 62)	49	13	79.0%	0.001
3% DQS (n = 62)	31	31	50.0%	
Refractive error < 0.50 D (n = 103)	73	30	70.9%	0.002
Refractive error ≥ 0.50 D (n = 21)	7	14	33.3%	

TBUT, tear break-up time; IPL-MGX, intense pulsed light–meibomian gland expression; DQS, diquafosol.

## Data Availability

The raw data supporting the conclusions of this study will be made available by the authors upon request. The data are not publicly available due to ethical restrictions.

## Abbreviations

The following abbreviations are used in this manuscript:

DQS	diquafosol sodium
IPL-MGX	intense pulsed light with meibomian gland expression
TBUT	tear break-up time
P-SE	postoperative absolute prediction error (spherical equivalent)
HOAs	higher-order aberrations
ROC	receiver operating characteristic
D	diopter
IOL	intraocular lens
DED	dry eye disease
MGD	meibomian gland dysfunction
AL	axial length
ACD	anterior chamber depthmean
K	mean keratometry
S-SE	subjective postoperative spherical equivalent
OCT	optical coherence tomography
OSDI	Ocular Surface Disease Index
CI	confidence interval
OR	odds ratio
GEE	generalized estimating equations
AUC	area under the curve
SD	standard deviation
IR	interquartile range
